# Spatial variation and predictors of anemia among women of reproductive age in Mozambique, 2022/23: a multiscale geographically weighted regression

**DOI:** 10.3389/fpubh.2025.1502177

**Published:** 2025-02-24

**Authors:** Deresse Abebe Gebrehana, Tadesse Tarik Tamir, Gebretsadik Endeshaw Molla, Yishak Kebede, Dejen Tegegne, Solomon Gedlu Nigatu, Araya Mesfin Nigatu

**Affiliations:** ^1^Department of Internal Medicine, School of Medicine, College of Medicine and Health Sciences, University of Gondar, Gondar, Ethiopia; ^2^Department of Pediatrics and Child Health Nursing, School of Nursing, College of Medicine and Health Sciences, University of Gondar, Gondar, Ethiopia; ^3^Department of Epidemiology and Biostatistics, Institute of Public Health, College of Medicine and Health Sciences, University of Gondar, Gondar, Ethiopia; ^4^Department of Health Informatics, Institute of Public Health, College of Medicine and Health Sciences, University of Gondar, Gondar, Ethiopia

**Keywords:** anemia, predictors, women, geographically weighted, Mozambique normal

## Abstract

**Introduction:**

Anemia is a critical global public health issue, especially among women of reproductive age (15–49 years) in low- and middle-income countries. Mozambique has the highest prevalence of anemia in women of reproductive age in Sub Saharan Africa in 2019. This study aims to assess the spatial variation and predictors of anemia among women of reproductive age in Mozambique.

**Methods:**

Individual record and spatial coordinates data from the Mozambique Demographic and Health Survey (DHS 2022/23) were used. A stratified two-stage cluster sampling method was applied. Global autocorrelation analysis was performed to determine clustering of anemia. A weighted sample of 5,907 women of reproductive age was analyzed using ArcGIS 10.7. Multiscale geographically weighted regression was employed to identify predictors of anemia.

**Results:**

The national prevalence of anemia among women of reproductive age in Mozambique was 51.89% (95% CI: 50.66, 53.12%). Higher rates were observed in Nampula, Zambezia, and Sofala. Unimproved drinking water, pregnancy, and being underweight were positively correlated with anemia, while contraceptive use and obesity were negatively correlated. Geographic variability in these associations was evident (Global Moran’s *I* = 0.0.444359 and *p* < 0.001). Anemia was the highest in Tete and Manica due to unimproved drinking water the effect of pregnancy on anemia was significant in Manica and Sofala. Being underweight was strongly related to anemia in Niassa. Conversely, contraceptive use and obesity had a protective effect in Nampula, Zambezia, Niassa, and Cabo Delgado.

**Conclusion:**

Anemia remains a critical public health issue among women of reproductive age in Mozambique, with marked regional disparities. Hotspot clusters were identified in Nampula, Zambezia, Tete, and Sofala. Factors such as unimproved drinking water, pregnancy, and being underweight were associated with higher anemia rates in certain regions, while obesity and contraceptive use indicated a protective effect in specific provinces. To effectively combat anemia, policymakers should focus on improving access to clean water and maternal health services, and enhancing nutritional support through the USAID Advancing Nutrition Project, the Global Alliance for Improved Nutrition, Integrated Community Case Management, and Supervised Weekly Iron and Folic Acid Supplementation.

## Introduction

Anemia, a condition characterized by a reduced concentration of hemoglobin in the blood (hemoglobin level < 12 g/dL and < 11 g/dL for non-pregnant and pregnant women respectively), is a global public health concern, particularly among women of reproductive age (WRA) in low- and middle-income countries (LMICs) (1). Anemia in WRA is associated with increased risks of maternal mortality, preterm birth, low birth weight, and impaired cognitive development in children (2, 3). According to the Ministry of Health (MISAU), improving nutritional support and addressing anemia among women is a priority in the National Health Policy and Strategic Plan 2022–2026 (4).

Worldwide, the prevalence of anemia among WRA is estimated to be around 30%, with the highest burden observed in Africa and Asia (1). In Mozambique, a country in Southeast Africa, anemia is a major nutritional deficiency problem, with an estimated prevalence of 55% among WRA as of 2019 (5). The burden of anemia is influenced by a variety of factors, with iron deficiency being the main cause (6). However, other micronutrient deficiencies (such as vitamin A, vitamin B12, and folate), chronic bleeding, acute or chronic infections, and parasitic infections (like hookworm and malaria) can also contribute to the development of anemia (7–9).

Estimates suggest that about half of anemia cases in low- and middle-income countries are attributable to iron deficiency, while the remaining cases may be due to diseases like parasitic infections, malaria, and HIV (2). A systematic review revealed that the proportion of anemia caused by iron deficiency was below 50% in LMICs, with regional variations, poor sanitary conditions, and increased occurrence of infections also contributing to the burden of anemia (10).

Spatial variation in anemia prevalence among women of reproductive age was observed in studies conducted in Ethiopia (11), Rwanda (12), and Nigeria (13). Understanding the spatial distribution and determinants of anemia in Mozambique is essential for developing targeted intervention strategies and allocating resources effectively. Previous studies have identified various factors associated with anemia in the country, including socioeconomic status, education level, access to healthcare, nutritional intake, and the burden of infectious diseases (14, 15). However, the geospatial patterns and clustering of anemia in WRA have not been comprehensively examined.

The Sustainable Development Goals (SDGs), specifically Goal 2: Zero Hunger, aim to end all forms of malnutrition, including anemia, by 2030 (16). The target is to reduce anemia among women of reproductive age by 50% by 2030 (16).

This study aimed to investigate the spatial distribution of anemia prevalence and identify the key determinant factors among WRA in Mozambique. By exploring the geographic distribution and analyzing the associated risk factors, the findings from this research can inform the development and implementation of tailored public health strategies to address anemia in this vulnerable population.

## Methods

### Study setting

**Figure 1:**
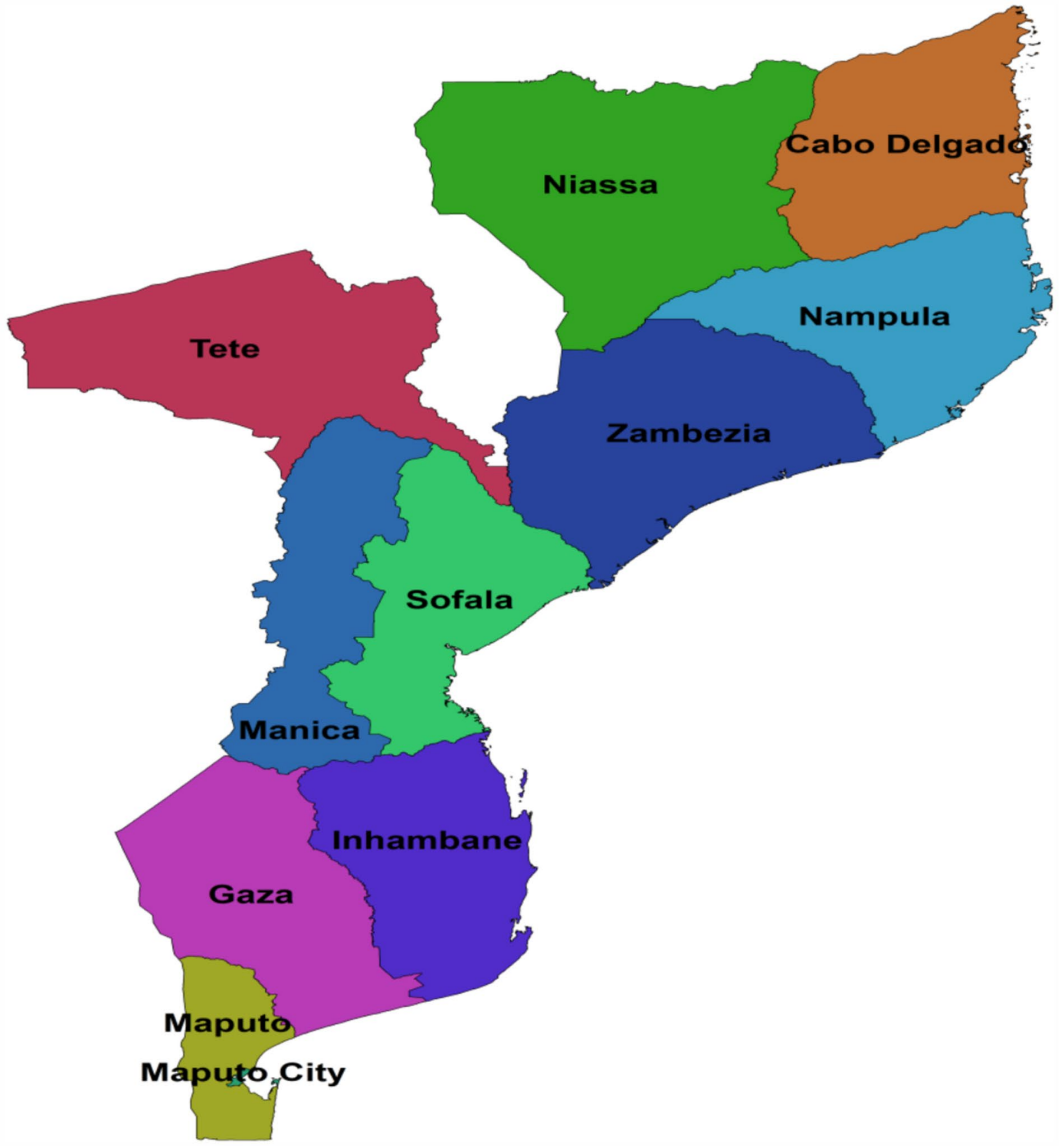
Graphical description of the study setting; Shape file source: https://data.humdata.org/dataset/5e8d83a5-1210-49be-b7d9-cf286dbc15df.

Mozambique is situated on the eastern coast of Southern Africa, facing Madagascar. It boasts approximately 2,800 km of coastline and covers an area of around 800,000 km^2^. Moving southward to Beira, the country consists of a large plain that lies 200–500 meters above sea level. To the west, the high plateaus of Zimbabwe, Swaziland, Natal, and Transvaal dominate the landscape. The central region features a high plateau nestled between Zambia and Zimbabwe along the Zambezi River. As we move eastward, Mozambique gradually descends toward the Indian Ocean, situated to the east of Lake Malawi. The country is divided into 3 regions (North, Center and South), 11 provinces and 148 districts (as shown in Figure 1). Maputo, the capital, is located in the southernmost part of the country. With a population estimated at slightly over 20 million, Mozambique experiences an annual growth rate of 2.5 percent. Agriculture plays a pivotal role in the country’s economy, employing more than 80 percent of the labor force and providing livelihoods for the majority of the population (17). Mozambique has a population density of 44 people per square kilometer (114 people per square mile). The current population of Mozambique is 35,063,881. The country’s total land area is 786,380 square kilometers (303,623 square miles). Approximately 41% of the population lives in urban areas (18). The north-central provinces of Nampula and Zambezia are the most populous regions in Mozambique. These provinces account for 45% of the total population. The largest city and capital, Maputo, has a population of around 1.2 million people (19).

### Study design and sample size

We conducted a cross-sectional study using data from the 2022/23 Mozambique Demographic Health Survey (DHS). The standard sampling technique used by DHS including the Mozambique 2022/23 involves a stratified two-stage cluster sampling approach to guarantee nationally representative data. First, a country is categorized into various strata based on relevant characteristics, such as urban or rural location and geographic regions. Within each stratum, enumeration areas (clusters) are randomly chosen as the primary sampling units, with these clusters typically containing several households. In the second stage, a sample of households is drawn from each selected enumeration area using either a systematic or random sampling method. This study focused on anemia among reproductive-age women within the 5 years preceding the survey. A total weighted sample of 5,907 reproductive age women. To analyze the data, we utilized individual recode (IR) data extracted from the Mozambique DHS 2022/23 data set (20). This can be accessed through: https://dhsprogram.com/data/dataset_admin/index.cfm.

### Variables of the study

The dependent variable of interest was anemia among women (15–49 years) in Mozambique. Anemia status was determined based on hemoglobin measurements collected as part of the 2022–23 Demographic and Health Survey (DHS) in Mozambique. As per the guide to DHS Statistics-8, individual non-pregnant women with a hemoglobin level of below 12 g/dL and pregnant women with hemoglobin level of less than 11 g/dL were classified as anemic and coded as “1,” otherwise non-anemic and coded as “0.”

Drawing from an extensive literature review, we examined several factors that may influence anemia in this population (21–27). These factors included maternal education, place of residence, maternal body mass index (BMI), maternal age, paternal education, marital status, maternal employment, use of antenatal care services, wealth status, family size, distance to health facilities, use of mosquito bed nets during sleep, media exposure, smoking status, type of toilet facility, source of drinking water, pregnancy status, use of contraceptive methods, breastfeeding status, sex of the household head, and parity (Table 1).

**Table 1:** Description of variables of the study.

Variable	Definition/categories
Age of the mother	The age of the mother was initially categorized as 15–19, 20–24, 25–29, 30–34, 35–39, 40–44, and 45–49. However, during data cleaning, we observed that some categories had very few participants, so we recoded the age groups to 15–24, 25–34, and 35–49.
Mother educational status	The education levels were coded as No-education, Primary, Secondary, and Higher in the EDHS dataset, and we used these categories as they were.
Marital status	Marital status was initially coded as never in union, married, living with partner, widowed, and divorced/separated. However, the number of participants in the last four categories was very low. Therefore, we combined married and living with partner into the category “in union,” and grouped the remaining categories into “not in union.
Place of residence	The data was coded as rural and urban, and we used these categories as they were
Pregnancy status	Pregnancy status was initially coded as no or unsure and yes. The no or unsure responses were recoded as not pregnant, and yes was recoded as pregnant.
House wealth index	In the MDHS, the household wealth index was categorized into quintiles: poorest, poor, average, rich, and richest. These categories were created using principal component analysis. For easier decision-making, we re-categorized the scale into three groups: poor, middle, and rich.
Use contraception method	MDHS initially coded the variable as No-method, Traditional method, and Modern method. However, we recoded it as No and Yes, by leaving No-method alone and combining Traditional and Modern methods.
Currently breastfeeding	Currently breastfeeding was coded as no and yes and we used it as it is.
Household size	Household size was from 1 to 23, the we categorized it in to One to two, Three to five and Six and above.
Slept under mosquito bed net	Slept under mosquito bed net was coded as yes and no, and we used as it is.
Body mass index	We divide the maternal body weight in kg by maternal height in meter square. Then BMI of <18.5, 18.5–24.9, 25–29.9, and ≥ 30 kg/m^2^ were coded as underweight, normal, overweight and obese, respectively.
Media exposure	Watching television, listening to radio and reading newspaper were added. Then media exposure was generated by classifying no listening to radio, no watching to television and no reading to newspaper as no media exposure and all others has media exposure.
Sex of household head	MDHS coded Sex of household head as male and female and we used as it is.
Distance to health facility	Distance to health facility was coded by MDHS as not big problem and big problem and we used as it is.
Smoking cigarettes	Smoking cigarettes was coded by MDHS as yes or no and we used as it is.
Parity	Total children ever born was ranged from 0 to 13 in the IR file of Mozambique DHS 2022/23. Then the variable parity was generated via recoding 0 as nulliparous, 1 as primiparous, 2–4 as multiparous and women having 5–13 children as grand multiparous.
Respondent currently working	Respondent currently working was coded in the DHS as yes or no and we used as it is.
Source of drinking water	Source of drinking water was coded in MDHS as piped into dwelling, piped to yard/plot, piped to neighbor, public tap/standpipe, tube well or borehole (with manual bomb), protected well (without manual bomb), unprotected well, protected spring, unprotected spring, river/dam/lake/ponds/stream/canal/irrig, rainwater, tanker truck, bottled water, other, and not a dejure resident. For our stud we categorized as piped into dwelling, piped to yard/plot, piped to neighbor, public tap/standpipe, tube well or borehole (with manual bomb), protected well (without manual bomb) protected spring, and rainwater as “improved” and all others as “unimproved.”
Type of toilet facility	Type of toilet facility was coded as flush to piped sewer system, flush to septic tank, flush to somewhere else, flush, do not know where ventilated improved pit latrine (vip), pit latrine with slab, pit latrine without slab/open pit, no facility/bush/field, no flush to piped sewer system, no flush to septic tank, no flush to somewhere else, no flush, do not know where, other, not a dejure resident. The categories flush to piped sewer system, flush to septic tank, flush to somewhere else, flush, do not know where, ventilated improved pit latrine (vip), pit latrine with slab were classified as improved toilet facility and all others were classified as unimproved toilet facility.

### Data management

The data were processed and cleaned using STATA version 17. This involved editing, verifying, and recoding the raw data as necessary (28). To account for the complex sampling design of the Mozambique Demographic and Health Survey (DHS), we generated a weighting variable. All subsequent analyses were conducted using the weighted data (29). This allowed us to restore the representativeness of the survey sample and obtain reliable statistical estimates. To explore the distribution of the dependent variable (anemia) and the independent variables, we generated cross-tabulations with the cluster variable (v001) and saved the results as comma-separated values (CSV) file in Excel. Then, we imported the data from Excel into ArcGIS 10.7 and fitted an ordinary least squares (OLS) regression model to further examine the spatial patterns and associations. The use of appropriate weighting and accounting for the complex survey design ensures the validity and generalizability of the study findings.

### Management of missing values

Women who were not tested, as well as those whose values were not recorded, were excluded from both the denominators and numerators.

### Statistical analysis

#### Spatial autocorrelation

The software Arc GIS version 10.7 was utilized to map model parameters between local models and look for spatial variance. Mozambique’s anemic distribution was determined by calculating the global spatial autocorrelation, also known as global Moran’s I (30, 31). A spatial statistic called Global Moran’s I uses the complete dataset to generate a single output value between −1 and +1. This allows for the measurement of spatial autocorrelation. A closer distance from −1 to Moran’s output suggests that the event of interest is scattered, whereas a closer distance from +1 suggests clustering, and a closer distance from 0 suggests a random pattern. The distribution of anemia among women of reproductive age is nonrandom, either clustered or scattered, according to a statistically significant Moran’s I (*p* < 0.05) (32).

#### Spatial interpolation

Based on measured values from the neighborhood, the standard Kriging method of spatial interpolation was used to forecast the percentage of anemia in un-sampled places. The Kriging method was preferred above other interpolation methods because an ideal interpolator that provides a minimum mean error (ME) and root mean square error (RMSE) is kriging interpolation (33).

#### Spatial scan statistical analysis

Bernoulli and merely spatial statistical analysis of Kulldorff’s scan were applied. Using SaTScanTM version 10.1.3 software, only regions with a high risk of prevalence were used to identify the geographic locations of statistically significant clusters of anemia. Because the data is binary (anemic or non-anemic), the Bernoulli model was applied (34). Anemia was classified as case (1) and its absence as non-case (0). To locate the important clusters, the coordinate (latitude and longitude) file, case file (1), and non-case file (0) were imported into the SaTScanTM program. A scale was used to determine the maximum scanning window size based on the proportion of the entire population at danger. The maximum size of a geographic cluster was set at 50% of the population at risk in order to account for both very tiny and very big clusters. Clusters that were most likely (primary) were found by means of the likelihood ratio and *p*-value tests. The most likely cluster is the one with the highest likelihood ratio (35).

### Factor analysis

The Ordinary Least Squares Model (OLS) is a global regression model that assumes the homogeneity of each variable’s coefficients throughout the study region and estimates the connection between the dependent and independent variables using a single equation (36). The initial step in selecting the suitable predictor variables for the geographical variation of anemia is to use the OLS model (37). Verification that the anemia does not have a stationary percentage is required before fitting the global and local regression models. Global spatial autocorrelation was used to determine the spatial no stationary. Global geographic regression modeling was then calibrated to find variables related to the percentage of anemia.

Before moving on to the local model, the six assumptions of the OLS model (that is, that the explanatory variables should have the relationship that we expected, that each explanatory variable is significant, that the residuals are random, that the Jarque-Bera statistics have no statistical significance, the VIF value, and the strength of R-square) were verified (38). The Variance Inflation Factor (VIF) values were used to evaluate the multicollinearity. Predictors with VIF values higher than 7.5, or the cut point to indicate that multicollinearity is present, were not seen in this set of data (28).

After verifying the assumptions of the OLS model, we employed the local model, Geographically Weighted Regression (GWR), to analyze spatially varying relationships at 95% confidence interval. GWR assumes that the relationships between variables change across space (39). We utilized the latest version of GWR, known as Multiscale Geographically Weighted Regression (MGWR) version 2.2.1 software. Unlike classic GWR, which assumes that all processes occur on the same spatial scale, MGWR allows for different processes to operate at varying scales (40). This advanced version avoids the single bandwidth assumption for all covariates, instead permitting covariate-specific bandwidths (40). Additionally, between local regression (MGWR) and global regression (OLS), corrected Akaike Information Criteria (AICc) and Adjusted *R*^2^ were used as model selection criteria. As the best fitted model, the one with the lowest AICc and the highest Adjusted *R*^2^ was chosen (37).

### Ethical consideration

Registration was completed and a permission letter was obtained in order to access the Mozambique 2022/23 DHS Dataset and Global Positioning System (GPS) data through the DHS measure website, http://www.dhsprogram.com/. As a result, all required information was obtained from the website of the Demographic and Health Surveys (DHS) Program. However, participant permission was not necessary since the authors used a supplementary dataset from the Mozambique DHS for 202/23.

## Results

### Characteristics of the study participants

In our study, a total weighted sample of 5,907 reproductive-age women with measured hemoglobin levels was included. Anemia was observed in 54.4% of women aged 15–19 years and 52.2% of women aged 35–49 years. Additionally, 54.5% of women residing in rural areas had anemia. The majority of women who were pregnant (60.6%), underweight (59.1%), poor (57.8%), or using unimproved sources of drinking water (59.6%) experienced anemia. Slightly more than half of the women with unimproved toilet facilities also had anemia (Table 2).

**Table 2:** Descriptive characteristics of the study participants (*n* = 5,907) in Mozambique DHS, 2022/23.

Variables	Anemia status			
	Anemic		Not anemic	
	Count	Percentage	Count	Percentage
Age of women in years				
15–19	749	54.4%	629	45.6%
20–34	1,093	49.7%	1,105	50.3%
35–49	1,218	52.2%	1,113	47.8%
Place of residence				
Urban	1,083	47.5%	1,195	52.5%
Rural	1,977	54.5%	1,652	45.5%
Type of toilet facility				
Improved	928	51.3%	881	48.7%
Unimproved	2,132	52.0%	1,966	48.0%
Educational level of the mother				
Not educated	902	56.8%	686	43.2%
Primary	1,347	53.6%	1,166	46.4%
Secondary	756	45.7%	899	54.3%
Higher	55	36.4%	96	63.6%
Use of contraception method				
No	2,512	56.6%	1,925	43.4%
Yes	548	37.3%	922	62.7%
Wealth index				
Poor	1,263	57.8%	923	42.2%
Middle	562	52.5%	508	47.5%
Rich	1,235	46.6%	1,416	53.4%
Current pregnancy status				
Not pregnant	2,789	51.1%	2,671	48.9%
Pregnant	271	60.6%	176	39.4%
Currently breastfeeding				
No	2,305	52.2%	2,107	47.8%
Yes	755	50.5%	740	49.5%
Household size				
One to two	198	57.9%	144	42.1%
Three to five	1,335	50.8%	1,293	49.2%
Six and above	1,527	52.0%	1,410	48.0%
Slept under mosquito bed net				
No	1,730	52.4%	1,572	47.6%
Yes	1,330	51.1%	1,275	48.9%
Body mass index				
Underweight	228	59.1%	158	40.9%
Normal	2,211	53.7%	1,906	46.3%
Overweight	441	46.2%	513	53.8%
Obese	180	40.0%	270	60.0%
Media exposure				
No exposure	1,692	56.7%	1,291	43.3%
Has exposure	1,368	46.8%	1,556	53.2%
Sex of household head				
Male	2,114	51.8%	1,971	48.2%
Female	946	51.9%	876	48.1%
Distance to health facility				
Not a big problem	1,734	48.5%	1,841	51.5%
Big problem	1,326	56.9%	1,006	43.1%
Source of drinking water				
Improved	1,759	47.2%	1,966	52.8%
Unimproved	1,301	59.6%	881	40.4%
Smoking cigarettes				
No	3,013	51.9%	2,795	48.1%
Yes	47	47.5%	52	52.5%
Current marital status				
In union	1,972	51.7%	1,846	48.3%
Not in union	1,088	52.1%	1,001	47.9%
Respondent currently working				
Working	784	44.8%	967	55.2%
Not-working	2,276	54.8%	1,880	45.2%
Parity				
Nulliparous	747	53.2%	656	46.8%
Primiparous	468	49.8%	472	50.2%
Multiparous	1,198	51.7%	1,119	48.3%
Grandmultiparous	647	51.8%	601	48.2%

### Prevalence of anemia among reproductive age women in Mozambique

**Figure 2:**
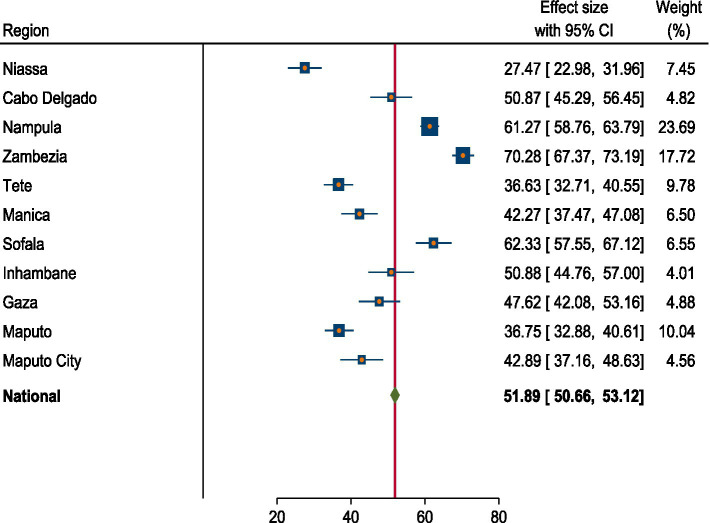
Forest plot of prevalence of anemia among women of reproductive age in Mozambique DHS, 2022/23.

The national pooled prevalence of anemia among women of reproductive age in Mozambique was 51.89% (95% CI: 50.66, 53.12%). Significant regional variations in anemia prevalence were observed. The highest prevalence was in Zambezia at 70.28% (95% CI: 67.37, 73.19%), while the lowest prevalence was in Niassa at 27.47% (95% CI: 22.98, 31.96%) (Figure 2).

### Spatial distribution of anemia among reproductive age women in Mozambique

**Figure 3:**
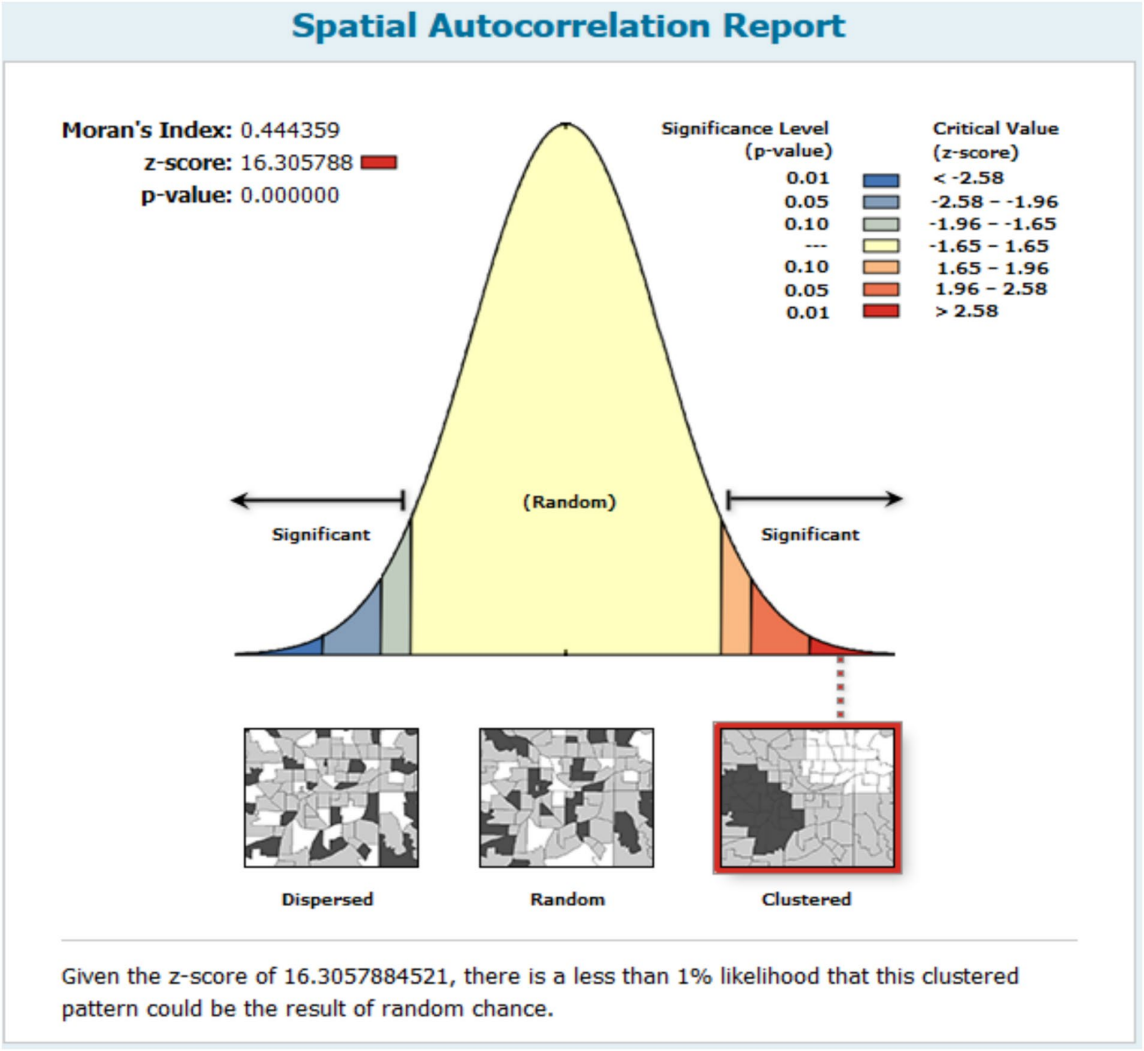
Global spatial autocorrelation result of anemia among reproductive age women in Mozambique DHS, 2022/23.

The spatial autocorrelation model found that the spatial distribution of anemia among reproductive-age women was non-random, with a global Moran’s I value of 0.444359 and a *p*-value of less than 0.001. This high confidence level indicates that the probability of the observed spatial pattern being due to randomness is less than 1%. The positive Moran’s I value signifies the clustering of anemia cases in Mozambique (Figure 3).

### Cluster and outlier analysis

**Figure 4:**
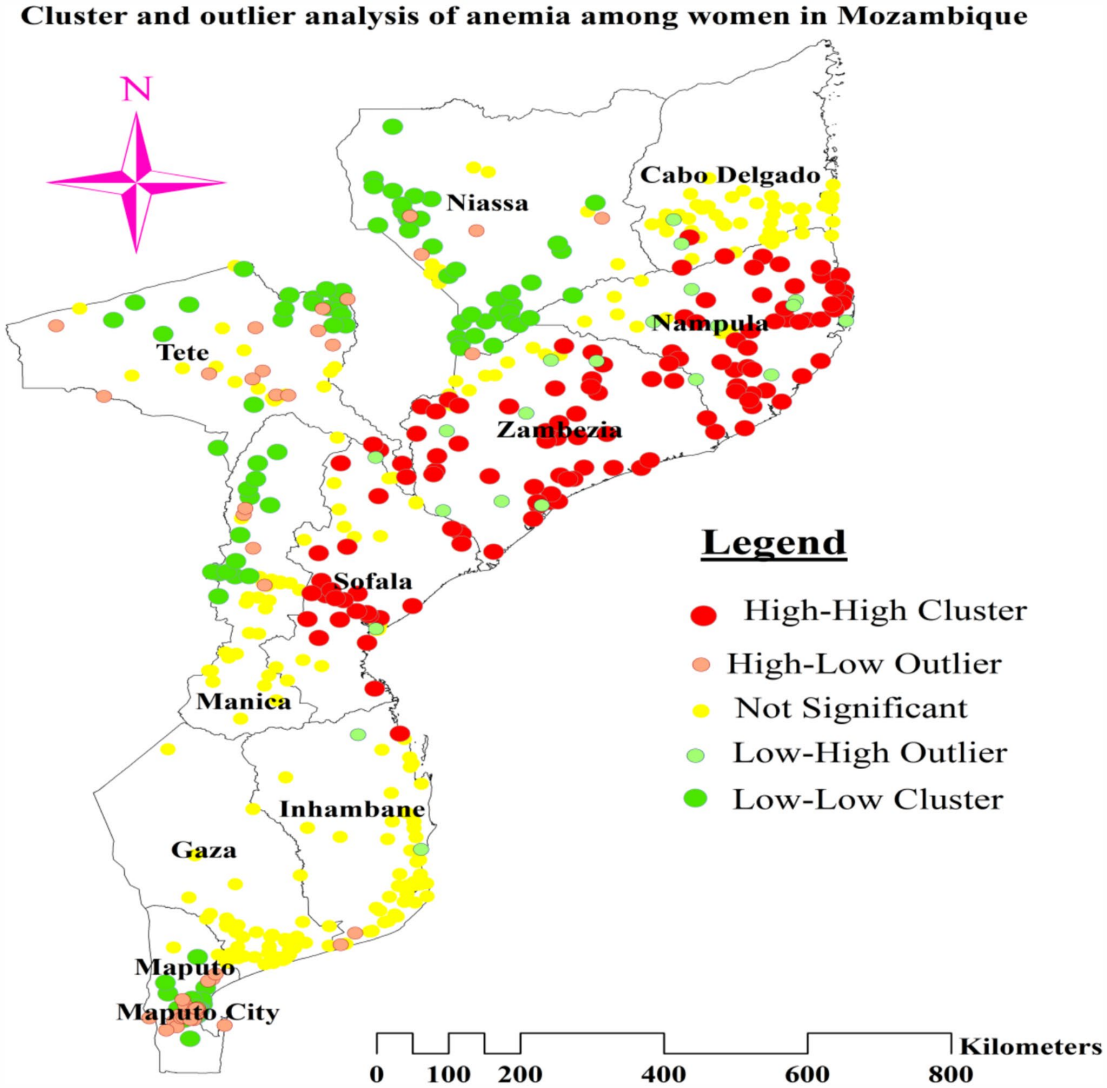
Cluster and outlier analysis of anemia among reproductive age women in Mozambique DHS, 2022/23.

**Figure 5:**
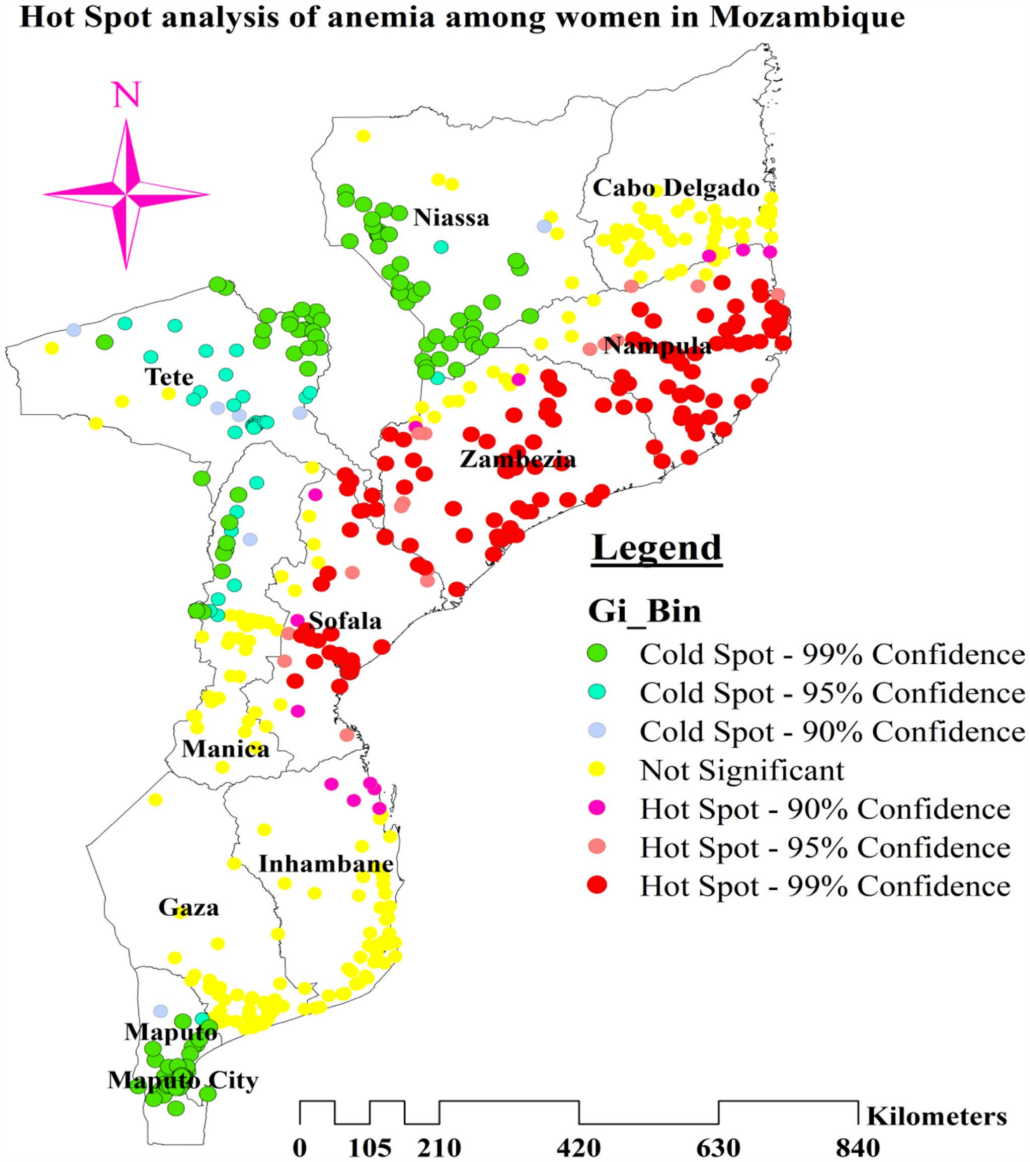
Hot Spot analysis of anemia among reproductive age women in Mozambique DHS, 2022/23.

We used the Anselin Local Moran’s I cluster and outlier analysis to illustrate the geographic distribution of anemia across the nation. This analysis identified specific clusters and their surrounding regions, as shown in Figure 4. The red tint in the panels indicates high-high clustering of anemia, signifying regions with a higher than average incidence of similar cases. These regions include most of Nampula, all of Zambezia, the northern and central areas of Sofala, and the eastern tip of Tete. Conversely, the northern half of Tete, the western and northwest boundaries of Manica, most of Maputo, and the northern section of Maputo City are low-low clustering zones, represented by green hues (Figure 5).

### Hotspot analysis of anemia among reproductive age women

The greater percentage of anemia (red dots) was centered in the majority of Nampula and Zambezia, the eastern tip of Tete, and the central and northern regions of Sofala. On the other hand, a lower proportion (represented by green dots) was found in the southern and southwest areas of Niassa, the northeastern part of Tete, a greater portion of Maputo, the northern section of Maputo City, and the Northwestern border of Manica (Figure 4).

### Spatial scan statistics of anemia among reproductive age women

Kulldorff’s spatial scan statistics identified three statistically significant clusters of anemia among reproductive-age women, using 50% of the total population as the maximum spatial circular window size. The most likely cluster had a total enumeration area of 101, a population size of 1,515, a number of cases of 1,047, and a LLR of 124.33. The primary cluster, centered at 17.269259 S and 37.822882 E, has a radius of 214.40 km, a relative risk of 1.51, and a *p*-value of less than 0.001. This reveals that women within this spatial window had a 51% elevated risk of anemia compared to women outside the window, and this risk was unlikely to have occurred by chance. The secondary cluster 1 (LLR = 105.69, *p*-value<0.001, and RR = 1.50) incorporated 79 enumeration areas, a population of 1,148, and 813 anemia cases among reproductive-age women. This first secondary cluster is centered at 17.269259 S, 37.822882 E, with a radius of 141.53 km. The population size and anemia cases in the scanning window of secondary cluster 2 were 841 and 540, respectively. It was located at 14.559803 S, 40.689502 E, with a 164.35 km radius. Its RR, LLR, and *p*-value were 1.29, 30.45, and <0.001, respectively. The third secondary cluster (total population = 74, anemia cases = 60, RR = 1.58, LLR = 13.91, and *p*-value <0.001) was centered at 19.339681 S, 34.339890 E, with a 51.93 km radius. This cluster with 12 enumeration areas is located in the central part of Sofala (Table 3).

**Table 3:** Cluster detection analysis results for anemia among reproductive age women in Mozambique DHS, 2022/23.

Cluster	*N*	Latitude	Longitude	Radius (Km)	Population	Cases	RR	LLR	*p*-value
Primary	101	17.269259 S	37.822882 E	281.87	1,515	1,047	1.51	124.33	<0.001
Secondary 1	79	17.269259 S	37.822882 E	252.73	1,148	813	1.50	105.69	<0.001
Secondary 2	48	14.559803 S	40.689502 E	164.35	841	540	1.29	30.45	<0.001
Secondary 3	12	19.339681 S	34.339890 E	51.93	74	60	1.58	13.91	<0.001

RR, relative risk; LLR, log-likelihood ratio; pop., population; N, Number of enumeration areas incorporated inside the scan window.

**Figure 6:**
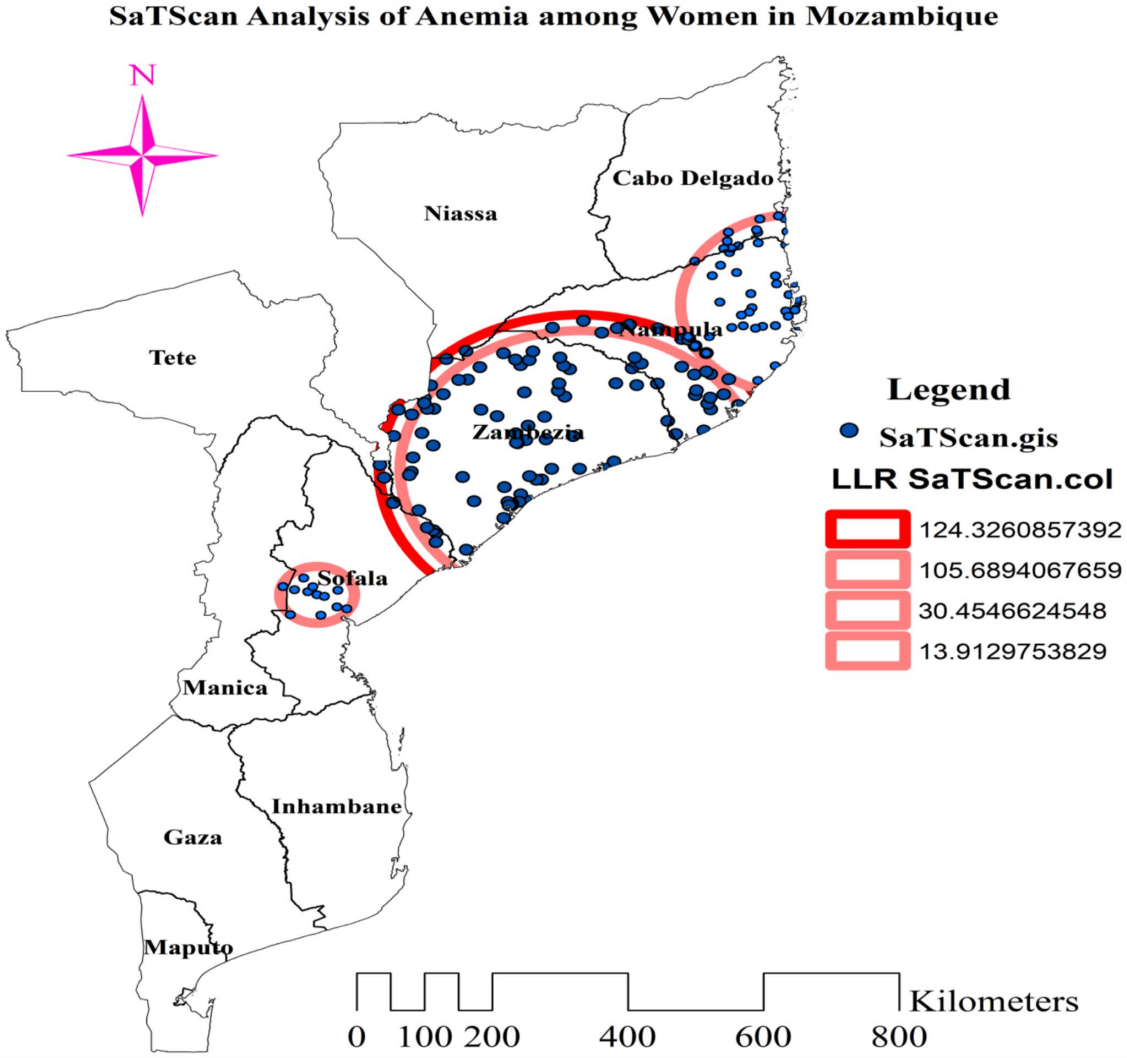
Most likely and secondary clusters with high rate of anemia among reproductive age women in Mozambique DHS, 2022/23.

In addition to the most likely cluster, three statistically significant secondary clusters were found in the western and northwestern parts of Mozambique, in the Nampula, Zambezia, and Sofala regions. The most likely cluster was concentrated in the whole of Zambezia, southern and southwestern Nampula, and northeastern Sofala. The secondary cluster 1 was situated in most parts of Zambezia, the northeastern border of Sofala, and southern and southwestern Nampula. The scanning window of secondary cluster 2 was situated in the eastern half of Nampula and the southeastern part of Cabo Delgado. The RR of 1.29 suggests that reproductive-age women within the spatial window had a 29% higher risk of anemia than women outside the window (Figure 6).

### Spatial interpolation of anemia among reproductive age women

**Figure 7:**
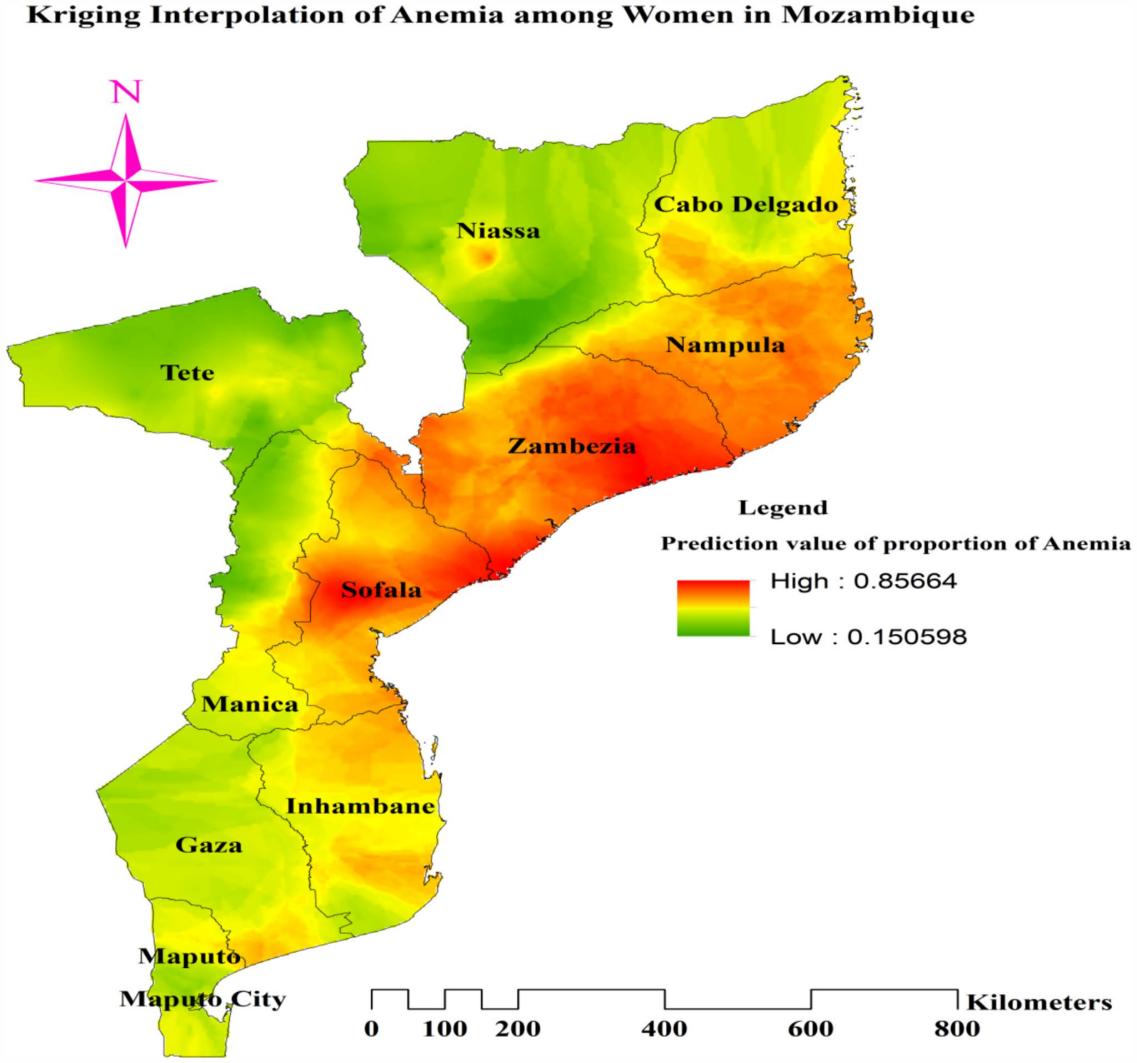
Ordinary Kriging of interpolation of proportion of anemia among reproductive age women in Mozambique DHS, 2022/23.

We mapped the prevalence of anemia among women of reproductive age across various regions in Mozambique using ordinary Kriging (OK). Higher proportions of predicted anemia (indicated by red shaded areas) were observed in nearly all of Nampula and Zambezia, the western, central, and eastern parts of Sofala, the southern portion of Cabo Delgado, the eastern tip of Tete, and a small area in central Niassa. Conversely, areas with green shading, which indicate expected low anemia prevalence, included most of Niassa, Tete, Manica, Cabo Delgado, and Gaza; the southern half of Maputo; and the southern and western borders of Inhambane (Figure 7).

### Spatial regression of the predictors of anemia

#### Ordinary least squares model results

We employed the OLS model to identify factors associated with spatial variation in anemia among reproductive-age women. Anemia was positively associated with using an unimproved drinking water source, being pregnant, and being underweight. Conversely, using contraceptive methods and being obese were negatively associated with anemia in this population. The minimum, maximum, and mean variance inflation factor (VIF) values were 1.61, 2.72, and 2.036, respectively, indicating the absence of multicollinearity among the independent variables used in the OLS model, as these values were below the cut-off threshold for multicollinearity diagnostics (Table 4).

**Table 4:** The ordinary least squares regression result summary.

Variables	Coefficients	Robust *t*-statistics	Robust probability	VIF
Intercept	0.510	30.53	0.000000*	–
Unimproved water source	0.009	4.16	0.000042*	2.72
Pregnancy	0.021	2.34	0.019654*	1.76
Use of contraception	−0.013	−2.66	0.007980*	2.33
Underweight woman	0.013	2.02	0.043658*	1.76
Obese woman	−0.019	−2.18	0.029908*	1.61

OLS diagnostics		
Diagnostic criteria	Magnitude	*p*-value
Number of data points	616	–
Adjusted R Squared	11.16%	–
AICc	1,712.38	–
Joint F-statistics	4.42	0.000000*
Joint Wald statistics	129.19	0.000000*
Koenker (BP) statistics	36.68	0.003723*
Jarque – Bera	4.61	0.099909

The adjusted *R*^2^ value of 0.1116 from the OLS model indicates that 11.165% of the variation in anemia is explained by the five explanatory variables (Table 4). This study demonstrates a statistically significant linear relationship between the dependent variable and the explanatory variables, as evidenced by significant joint Wald and F-statistics. The *p*-value of 0.099909 for the Jarque-Bera statistic suggests that the residuals are normally distributed, indicating that the OLS model predictions are unbiased. However, the statistically significant Koenker statistic indicates that the regression model is inconsistent across the study area, suggesting that the relationship between variables varies with geographic location. Consequently, the GWR model was deemed more appropriate and was used to estimate the model parameters (Table 4).

### Multiscale geographically weighted regression analysis

Comparing the OLS model and the Multiscale Geographically Weighted Regression (MGWR) model using diagnostic parameters (AICc and *R*^2^), the AICc value decreased from 1712.38 (OLS model) to 1538.93 (MGWR model). The adjusted *R*^2^ value increased from 0.116 (11.16%) in the OLS model to 0.442 (44.2%) in the MGWR model. These diagnostic parameters indicate that the MGWR model is superior to the OLS model, suggesting that the Multiscale Geographically Weighted Regression (local) model provides a better fit than the OLS (global) model (Table 5).

**Table 5:** Model comparison of OLS and MGWR model fit/performance of anemia among reproductive age women in Mozambique DHS, 2022/23.

Fitness parameter	Global model	MGWR model
AICc	1,712.38	1,538.93
R-squared	0.112 (11.2%)	0.442 (44.2%)
Adjusted R-squared	0.088 (8.8%)	0.375 (37.5%)

The mean and median beta coefficients for using an unimproved water source, being pregnant, and being underweight were positive, indicating a positive association between these factors and the spatial variation of anemia. Conversely, the mean and median beta coefficients for using contraceptive methods and being obese were negative, suggesting a negative association between these factors and the spatial variation of anemia (Table 6).

**Table 6:** Summary statistics for MGWR parameter estimates describing the spatially varying relationships between anemia among reproductive age and predictors in Mozambique DHS, 2022/23.

Variable	Mean	STD	Min	Median	Max
Intercept	0.013	0.498	−1.300	−0.004	1.138
Unimproved water source	0.193	0.017	0.162	0.205	0.210
Pregnancy	0.114	0.004	0.109	0.113	0.125
Use of contraception	−0.123	0.024	−0.147	−0.137	−0.088
Underweight	0.103	0.023	0.079	0.098	0.165
Obese	−0.099	0.023	−0.131	−0.096	−0.073

**Figure 8:**
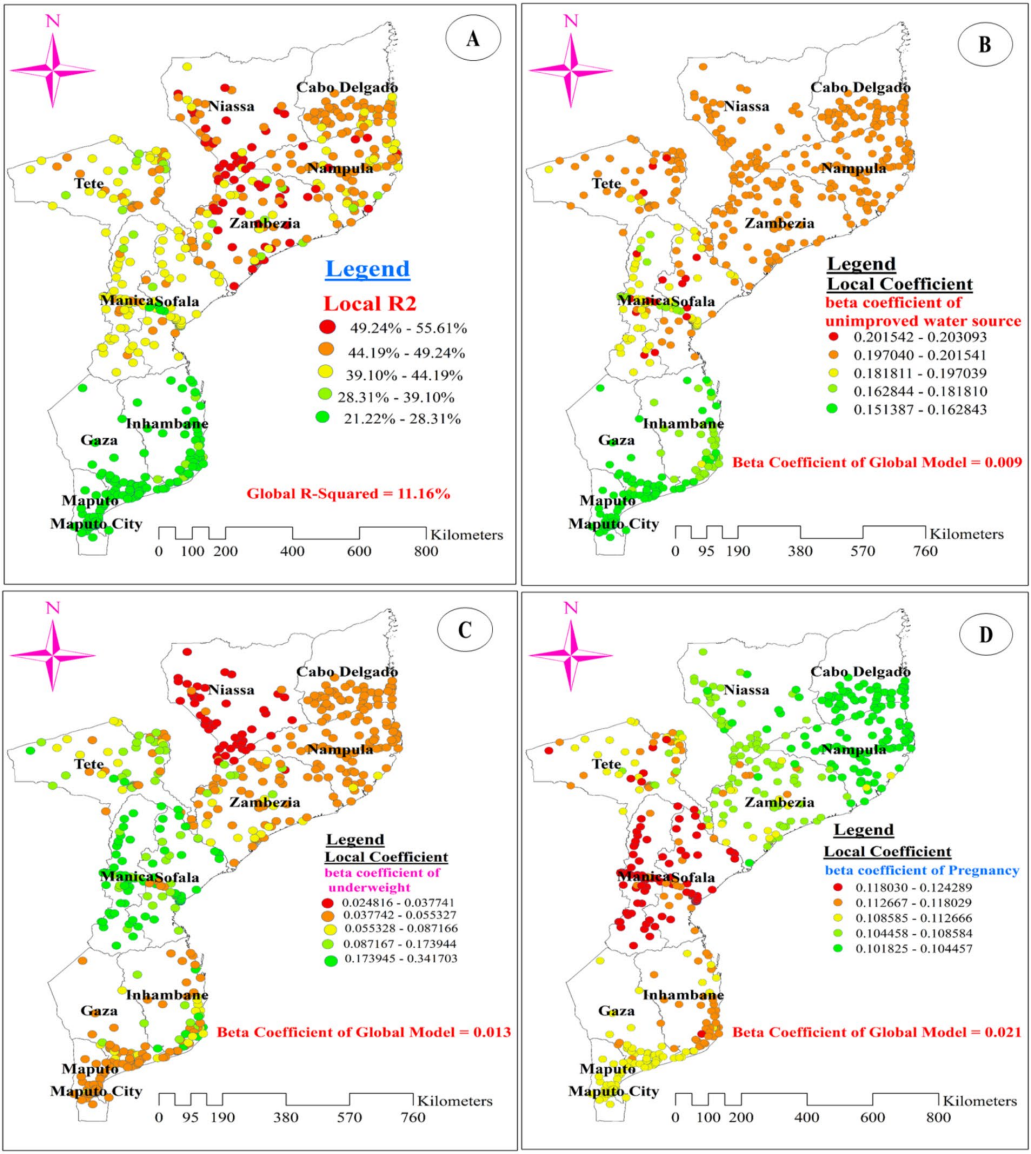
The spatial mapping of local R-squared **(A)** and local regression coefficients of unimproved drinking water source **(B)**, underweight **(C)**, and pregnancy **(D)** in Mozambique DHS, 2022/23.

Figure 8A illustrates the model’s performance (local R-squared) across the study area. The model performed well, with R-squared values ranging from 46.28 to 55.61% in the entire areas of Zambezia, southern, southeastern, and southwestern Niassa, and the northern border of Nampula. However, the model showed a poor fit for data from provinces in the south, southwest, and southeast of Maputo, the north, northeast, and northwest of Maputo City, the south of Gaza, and the east, southeast, and northeast of Inhambane, with adjusted R-squared values ranging from 21.22 to 28.31% (Figure 8A).

**Figure 9:**
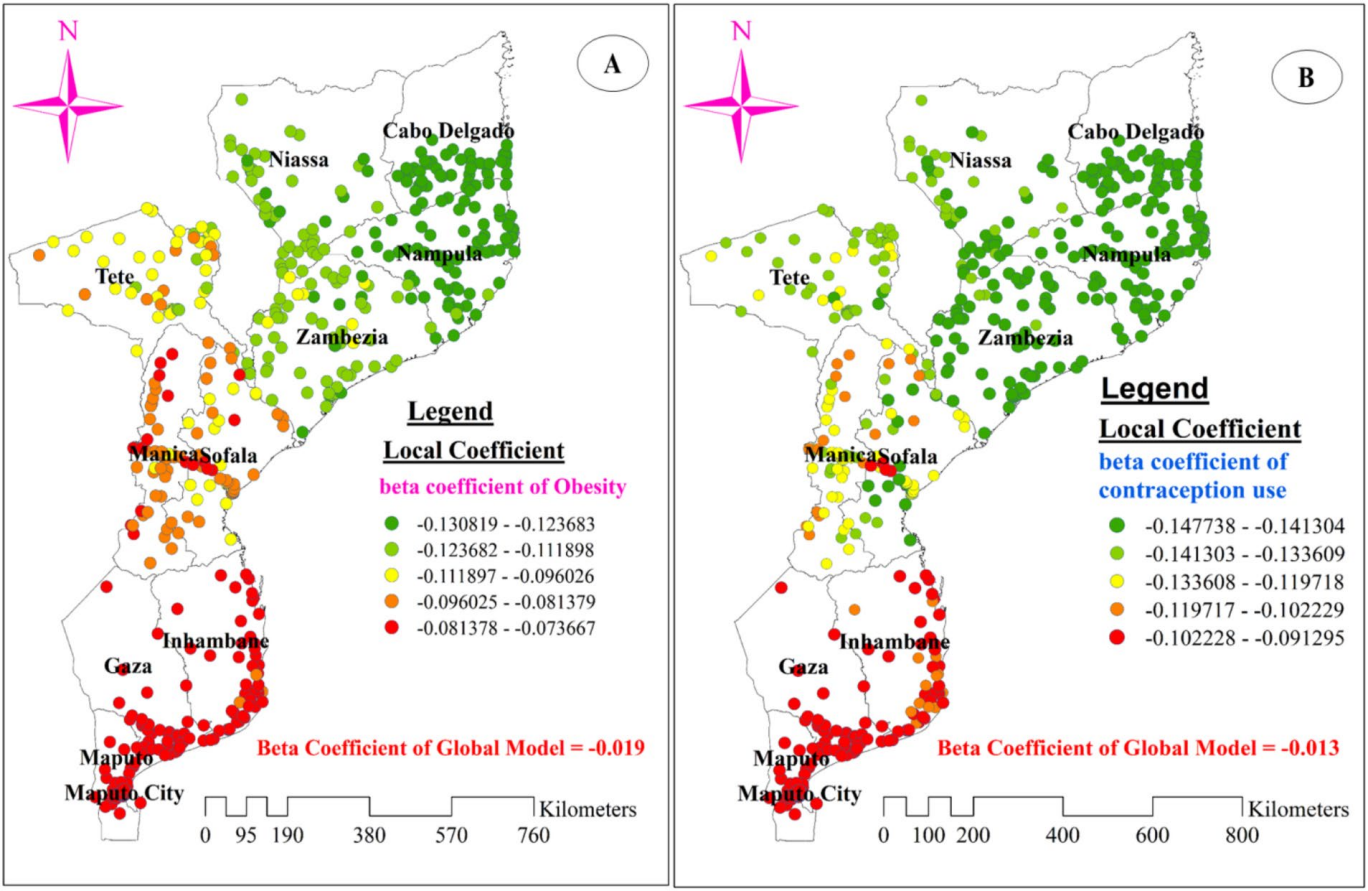
The spatial mapping of local regression coefficients of obesity **(A)**, and use of contraception methods **(B)** in Mozambique DHS, 2022/23.

Figures 8B–D, 9A,B display the spatial distribution of the beta coefficients for the five predictor variables. The red dotted areas indicate a strong positive influence (high beta coefficient) of three explanatory variables (using an unimproved drinking water source, being pregnant, and being underweight) on anemia among reproductive-age women (Figures 8B–D). Conversely, the green dotted areas show a strong negative influence (high protective effect) of two explanatory variables (using contraceptive methods and being obese) on anemia among reproductive-age women (Figures 9A,B).

The beta coefficient for using an unimproved drinking water source shows substantial variation across the study area, indicating an inconsistent relationship between this variable and the proportion of anemia. A strong positive influence of using an unimproved drinking water source on anemia was observed in the northeast of Tete, central and southeast Manica, and the west and central parts of Sofala. Conversely, the lowest coefficients for women using an unimproved drinking water source were predominantly found in southern Gaza, southern Maputo, and northern Maputo City. Additionally, low coefficients were scattered across the central part of Gaza and the northern, eastern, and southern borders of Inhambane (Figure 8B).

Regarding the beta coefficient for pregnant women, a strong positive influence (red dotted areas on the map) was concentrated in most parts of Manica and Sofala. Although scattered, it was also observed in the southeast and northeast parts of Tete, with beta coefficient values ranging from 0.118 to 0.124 in these areas. Conversely, the lowest coefficients (green dotted areas on the map) for pregnant women were predominantly found in the entire area of Nampula and the southern part of Cabo Delgado (Figure 8D). Additionally, being underweight was associated with spatial variation in anemia. A strong positive relationship (red dotted areas) between being underweight and anemia was identified in most parts of Niassa, particularly in the southern and western regions of the province. The beta coefficients in these areas ranged from 0.025 to 0.038. Conversely, low beta coefficients were notably observed in most parts of Manica and Sofala (Figure 8C).

Interestingly, the negative beta coefficients for using contraceptive methods and being obese imply a protective effect of these explanatory variables on anemia among reproductive-age women. A strong protective effect, indicated by green-dotted areas on the map, was concentrated in all parts of Nampula and the southern part of Cabo Delgado. This effect was also observed in Zambezia (especially for using contraceptive methods) and the eastern and western portions of Niassa (Figures 9A,B).

## Discussion

Mozambique, one of the countries with the highest prevalence of anemia in sub-Saharan Africa, was the focus of this study, which identified the prevalence, predictors, and spatial variation of anemia among women of reproductive age (5).

The national pooled prevalence of anemia among women of reproductive age in this study was 51.89% (95% CI: 50.66, 53.12%). This finding aligns with the prevalence reported in the National Family Health Survey (NFHS)-4 in India, which stands at 53% (41).

However, it is higher than the prevalence reported in studies conducted in Rwanda (19.2%) (24), Sudan (35.6%) (42), and China (18.9%) (43). These significant differences in anemia prevalence can be attributed to variations in healthcare access, nutrition, public health interventions, cultural beliefs, food insecurity and socio-economic status. In Mozambique, frequent natural disasters such as cyclones, floods, and droughts disrupt livelihoods and food production, exacerbating the challenges faced by vulnerable populations and thereby increasing the prevalence of anemia among women of reproductive age (44). Despite ongoing interventions in Mozambique such as the USAID Advancing Nutrition Project (45), the Global Alliance for Improved Nutrition (GAIN) (46), the prevalence of anemia in women of reproductive age is still higher. In China, the low prevalence of anemia can be attributed to a more intensive and geographically extensive multifaceted approach that includes Iron and Folic Acid Supplementation, Dietary Diversification, and Nutrition Counseling and Education (47, 48). In Rwanda, socio-economic indicators such as literacy rates and poverty levels are generally better than those in Mozambique (49, 50), contributing to the lower prevalence of anemia. In Sudan, the relatively better Malaria Control, Healthcare Access, Nutritional Interventions, and Public Health Programs compared to in Mozambique contribute the lower prevalence of anemia (51).

Conversely, the prevalence of anemia in this study is slightly lower than the national prevalence in Yemen, which stands at 57.4% (27). The slightly lower prevalence compared to Yemen may be due to differences in healthcare access, nutritional programs, and public health interventions. In Yemen, the higher prevalence of anemia can be attributed to ongoing conflict, food insecurity, and restricted access to healthcare facilities (52, 53). These factors increase the risk of anemia, especially for women of reproductive age who require more iron because of menstruation, pregnancy, and lactation (54, 55).

The results from the spatial global Moran’s analysis in our study illustrate that the proportion of anemia among reproductive age women in Mozambique demonstrate significant geographical variation and clustering. This finding is supported by similar studies conducted in Democratic republic of Congo (DRC), Ethiopia, Timore-Leste, India, and Bangladesh which showed considerable clustering and high prevalence of anemia in particular areas of the countries (25, 41, 56–58).

Considering the explanatory variables, the spatial clustering of anemia can be attributed to various underlying factors that are geographically concentrated. These factors include poverty, illiteracy, poor sanitation, malnutrition, insufficient regional health services, inadequate dietary practices like low dietary diversity and poor meal frequency, limited nutritional supplementation like insufficient coverage of supplementation programs and challenges in implementation, limited healthcare access (including antenatal care), recent childbirth, and the incidence of malaria (25, 41, 57). Additionally, maternal age, pregnancy status, body mass index, and HIV status were significant predictors for the geographical variation of anemia among reproductive age women (56, 58).

This study’s spatial analysis revealed distinct geographical patterns in the prevalence of anemia among women of reproductive age in Mozambique. The hotspot analysis identified several regions with significantly higher proportions of anemia, particularly in Nampula, Zambezia, Tete, and Sofala. These hotspots can be attributed to a combination of factors, including nutritional deficiencies, socioeconomic conditions, infectious diseases, and natural disasters (notably in Nampula and Sofala). Additionally, reproductive health factors such as access to reproductive health services, HIV/AIDS, and sexually transmitted infections (STIs) contribute to the high prevalence of anemia in these provinces. Firstly, the availability of a balanced diet high in iron, folate, and vitamin B12—all necessary for the synthesis of healthy red blood cells—is restricted for many women in these areas (59). Secondly, the high prevalence of parasitic infections such as malaria and hookworm resulting in hemolysis and chronic blood loss, respectively, is another significant factor, leading to anemia (60, 61). Thirdly, many women cannot afford or access the essential treatments and preventative measures for anemia due to high levels of poverty and restricted access to healthcare facilities (62). Finally, Short time spans between pregnancies and repeated pregnancies can deplete a woman’s iron levels, enhancing the risk of anemia (63).

On the other hand, a lower proportion was found in Niassa, Tete, Maputo, Maputo City, and Manica. The relatively lower proportion of maternal anemia in these specific areas of Mozambique might be attributed to several factors like healthcare access and quality, malaria control, nutritional programs, higher socioeconomic status, and higher levels of education and awareness about maternal health. For example, compared to more remote areas, places like Maputo and Maputo City have better access to medical services and a stronger healthcare infrastructure. Better prenatal care is one aspect of this, since it can aid in the early diagnosis and treatment of anemia (64).

The GWR analysis revealed substantial spatial variation in the relationship between using an unimproved drinking water source and the proportion of anemia among reproductive women across Mozambique. This suggests that the impact of water quality on anemia prevalence is highly context-dependent and cannot be adequately captured by a single, global model (65, 66). Specifically, the GWR results showed a strong positive influence of using an unimproved water source on anemia levels in specific regions of the country, namely the northeast of Tete, central and southeast Manica, and the west and central parts of Sofala. In these areas, women who rely on unimproved water sources, such as surface water, unprotected wells, or unprotected springs, are at a significantly higher risk of being anemic compared to those with access to improved water sources. This aligns with finding from previous study in low and middle income countries in 2011 (2). This spatial variation in the water source-anemia relationship suggests that there are likely other contextual factors, such as local environmental conditions, sociocultural practices, or access to health services that modify the impact of water quality on anemia prevalence (67, 68). These factors may differ across the regions of Mozambique, leading to the observed heterogeneity in the GWR model results (65, 66).

Additionally, being pregnant was significantly related to anemia, with its stronger positive influence concentrated in most parts of Manica, Sofala, southeast and northeast parts of Tete. This finding aligns with evidence from previous studies (69–73). The observed spatial variation in the pregnancy and anemia relationship can be attributed to a combination of physiological, access to care, socioeconomic, and environmental factors. Physiological factors such as maternal nutritional status and underlying health conditions can contribute to the risk of anemia during pregnancy (69, 70). Access to quality antenatal care, including regular checkups and appropriate supplementation, has also been shown to play a crucial role in mitigating anemia prevalence (71, 72). Socioeconomic factors, such as household wealth and education levels, significantly influence the spatial distribution of anemia among pregnant women. Women from lower socioeconomic backgrounds often have limited access to nutritious food, healthcare services, and health education, which can lead to higher rates of anemia. Additionally, cultural determinants, such as traditional dietary practices and beliefs about health and illness, also play a crucial role. For example, certain cultural practices may restrict the consumption of iron-rich foods, contributing to higher anemia prevalence (73, 74). Additionally, environmental exposures, such as the availability of iron-rich foods and the presence of infectious diseases, can also contribute to the observed spatial variation in the pregnancy-anemia relationship (75, 76).

Moreover, aligning with existing evidence (71, 72, 75, 76), being underweight was associated with spatial variation in anemia. A stronger positive relationship between being underweight and anemia was identified in Niassa. This finding suggests that maternal nutritional status, as indicated by underweight, is an important determinant of the spatial distribution of anemia among reproductive age pregnant women. Underweight women are more likely to have inadequate nutrient intake, which can directly contribute to the development of anemia during pregnancy (70, 77). Additionally, underlying health conditions related to underweight, such as chronic infections or malabsorption disorders, can further exacerbate the risk of anemia (71, 72). The spatial clustering of the underweight-anemia relationship in Niassa may be influenced by socioeconomic factors, such as poverty and food insecurity, which can limit access to a diverse and nutrient-rich diet (73, 74). The availability of iron-rich foods and the presence of infectious diseases, may also contribute to the observed spatial patterns (75, 76). Addressing the underlying causes of underweight and improving maternal nutritional status through Integrated Community Case Management (iCCM) (78) and Supervised Weekly Iron and Folic Acid Supplementation (WIFS) (79) could be crucial interventions for reducing the spatial disparities in anemia prevalence among pregnant women in the region. Furthermore, our analysis revealed that using contraceptive methods was inversely related with anemia among women in Mozambique, with a stronger protective effect concentrated in Nampula, Zambezia, Cabo Delgado and Niassa provinces. This finding suggests that access to and utilization of contraceptive services may play a crucial role in mitigating the burden of anemia among women of reproductive age in these regions. The observed spatial variation in the contraceptive use-anemia relationship can be attributed to several factors. First, the availability and quality of family planning services, including the provision of a diverse range of contraceptive methods, may be more robust in the identified provinces compared to other areas of the country. Improved access to contraception can empower women to plan their pregnancies, which is particularly important given the strong association between pregnancy and anemia risk (75, 76). Additionally, the use of certain contraceptive methods, such as intrauterine devices or implants, can help prevent frequent or closely spaced pregnancies, reducing the physiological strain that repetitive pregnancies can have on a woman’s iron stores and overall nutritional status (80, 81).

The GWR analysis also revealed an intriguing finding - being obese was inversely related to anemia among women in Mozambique, with a stronger protective effect in Nampula, Cabo Delgado, and Niassa provinces. This inverse association between obesity and anemia prevalence within these regions merits further study. However, this finding may be explained by the notion that these regions of Mozambique may have stronger health infrastructure and services, including more effective anemia screening, diagnosis, and management programs that target both overweight/obese and underweight individuals (68, 82, 83). Furthermore, improved roads on access to health care, addressing health care disruption in rural Mozambique could be the potential explanation (84, 85). Additionally, the interplay between obesity, inflammation, and anemia should be considered. Obesity is often associated with chronic low-grade inflammation, which can lead to the development of functional iron deficiency and anemia of inflammation (86, 87). However, in certain contexts, the inflammatory response may be less pronounced or better managed, allowing obese individuals to maintain adequate iron status and hemoglobin levels. Further research is needed to unpack the complex mechanisms underlying the spatial patterns of the obesity-anemia relationship in Mozambique.

## Conclusion

Anemia remained a critical public health issue among women of reproductive age in Mozambique, with marked regional disparities. Hotspot clusters of anemia were found in Nampula, Zambezia, Tete, and Sofala, while cold spot clusters were located in Niassa, Tete, Maputo, Maputo City, and Manica. Factors such as unimproved drinking water, pregnancy, and being underweight were associated with higher anemia rates in certain regions, whereas obesity and contraceptive use indicated a protective effect in specific provinces. Targeted interventions addressing these factors are essential for reducing anemia and improving health outcomes for women across Mozambique; therefore, policymakers should focus on improving access to clean water, increasing the availability of maternal health services and enhancing nutritional support through the USAID Advancing Nutrition Project, the Global Alliance for Improved Nutrition (GAIN), Integrated Community Case Management (iCCM), and Supervised Weekly Iron and Folic Acid Supplementation (WIFS), particularly in the identified hotspot regions.

### Limitation

The study’s conclusions should be interpreted with the following limitations in mind: Firstly, due to its cross-sectional design, the study cannot establish the chronological order of events, which restricts our ability to make causal inferences about anemia predictors. Secondly, the study relied on secondary data from Mozambique’s Demographic and Health Survey (DHS), which did not account for genetic causes of anemia such as iron-refractory iron-deficiency anemia, sickle cell anemia, thalassemia, and hereditary spherocytosis. These genetic factors could potentially influence anemia risk. Additionally, the study’s narrow focus on Mozambique limits the generalizability of findings to other regions with varying healthcare access, nutrition programs, public health interventions, socio-economic status, and cultural practices. Lastly, the lack of information on anemia type—due to the absence of peripheral morphology assessment and iron panel studies (including ferritin, serum iron, transferrin saturation, and total iron binding capacity), serum vitamin B12 levels, and serum folate levels—poses challenges in characterizing the specific anemia subtypes.

## Data Availability

Publicly available datasets were analyzed in this study. This data can be found here: https://www.dhsprogram.com/data/available-datasets.cfm.
